# Primary Cutaneous Diffuse Large B-cell Lymphoma, Leg Type, With Rapid Systemic Progression in an Elderly Patient: A Case Report

**DOI:** 10.7759/cureus.117174

**Published:** 2026-09-29

**Authors:** Amara Hazel Solorio Rivera, María Fernanda Limon Limon, Bryan Salvador Sol, Andrea Sierra Franco, Edgar Alejandro Sanroman Loza

**Affiliations:** 1 Dermatology, Instituto Dermatológico de Jalisco "Dr. José Barba Rubio", Guadalajara, MEX

**Keywords:** b-cell lymphoma, cutaneous lymphoma, leg type, primary cutaneous b-cell lymphoma, primary cutaneous diffuse large b-cell lymphoma, skin neoplasms

## Abstract

Primary cutaneous diffuse large B-cell lymphoma, leg type (PCDLBCL-LT), is an aggressive neoplasm characterized by a high proliferative rate and a tendency for systemic dissemination. It typically presents in older adults as erythematous nodules or plaques on the lower extremities. We report the case of a 77-year-old man with a nodular lesion on the right leg of seven months’ duration, which progressed to multiple confluent nodules forming a plaque with necrotic areas. Histopathological examination revealed a diffuse infiltrate of large cells expressing CD20, BCL-2, and MUM-1, with a high Ki-67 proliferation index. Based on the clinical, histopathological, immunohistochemical, and staging findings, a diagnosis of PCDLBCL-LT, stage T2aN0M0, was established. Following six cycles of R-CHOP immunochemotherapy and consolidative radiotherapy, the patient developed disease progression with bone marrow involvement and progression to systemic disease. This case highlights the aggressive clinical behavior of this entity and the need for close follow-up.

## Introduction

Primary cutaneous diffuse large B-cell lymphoma, leg type (PCDLBCL-LT), is a rare, aggressive primary cutaneous B-cell lymphoma that accounts for about 4% of primary cutaneous lymphomas [1]. It predominantly affects elderly women and presents as erythematous nodules on the lower extremities [2-4]. Histologically, it is characterized by a diffuse infiltrate of immunoblasts and centroblasts expressing CD20, BCL-2, and MUM-1 [2,5]. Its pathogenesis involves recurrent molecular alterations affecting B-cell receptor and NF-κB signaling pathways. Mutations involving MYD88, CD79B, CARD11, and TNFAIP3/A20 contribute to constitutive NF-κB activation and may underlie its aggressive biological behavior [1]. It is associated with aggressive behavior, with a five-year survival rate ranging from 41% to 66% [3,4]. Standard treatment includes rituximab-based immunochemotherapy (R-CHOP) [3]. Given the rarity of PCDLBCL-LT and its potential for aggressive extracutaneous dissemination, we report a case in a male patient who developed bone marrow involvement only two months after completing R-CHOP, indicating the potential for early systemic progression despite standard immunochemotherapy.

## Case presentation

A 77-year-old man with a past medical history significant for type 2 diabetes mellitus and atrial fibrillation, on treatment with rivaroxaban, presented with a cutaneous lesion on the right leg of approximately seven months’ duration. The patient reported that the lesion initially appeared as a small erythematous nodule that progressively increased in size and remained asymptomatic. He denied associated systemic symptoms.

Physical examination revealed multiple erythematous, infiltrated nodules, some confluent, forming a tumoral plaque measuring approximately 16 × 6 × 0.5 cm in the pretibial region of the right leg, with areas of ulceration and superficial necrosis. The lesion had poorly defined borders and a firm consistency on palpation (Figure 1).

**Figure 1:**
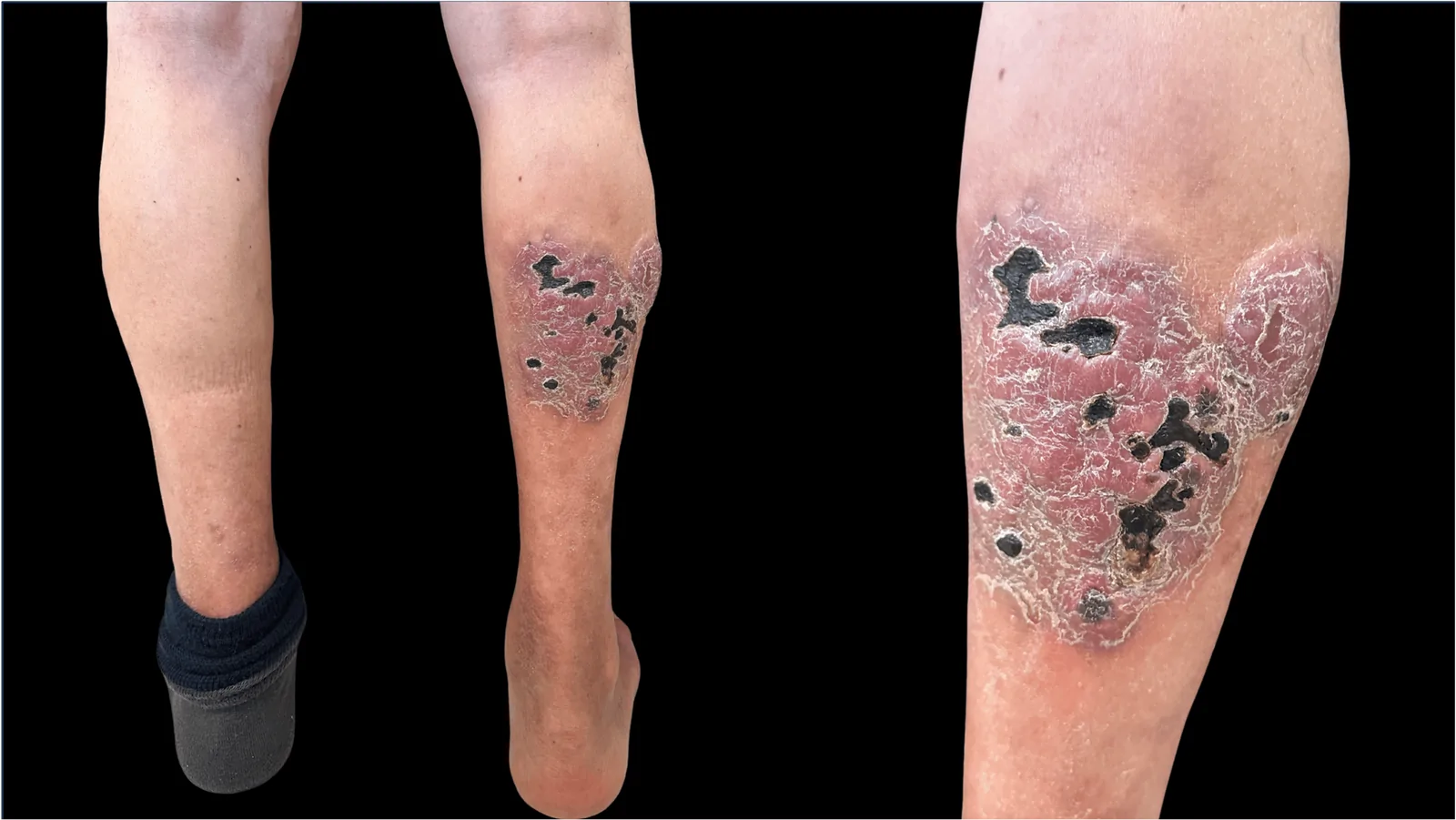
Nodules coalescing into a plaque with areas of necrosis on the surface

No palpable lymphadenopathy was identified in regional nodal chains. An incisional biopsy of the cutaneous lesion was performed. Histopathological examination demonstrated a diffuse dermal infiltrate composed of large lymphoid cells with vesicular nuclei and prominent nucleoli, sparing the epidermis (Figure 2A-2B).

**Figure 2:**
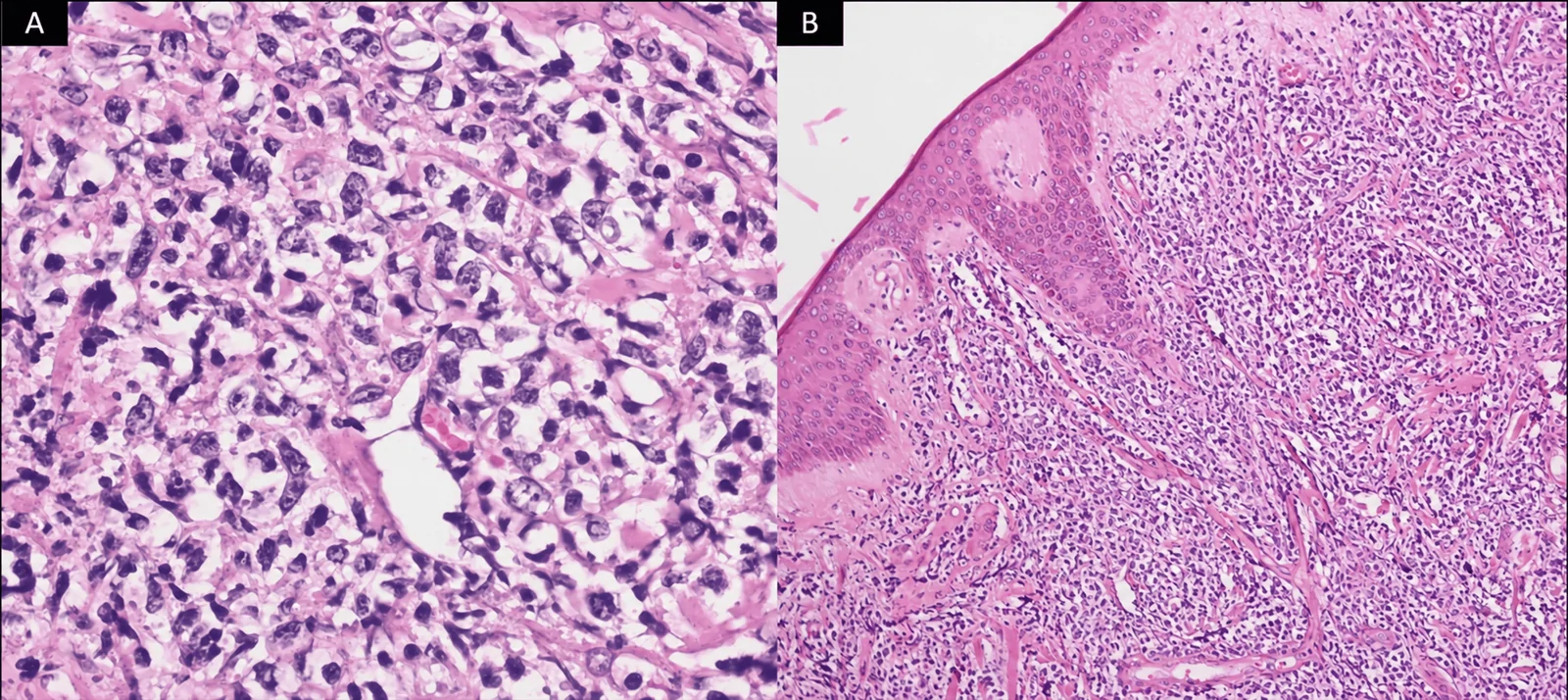
(A) High-power view showing large lymphoid cells with vesicular nuclei and prominent nucleoli (H&E ×400). (B) Diffuse dermal infiltrate of large atypical lymphoid cells with sparing of the epidermis (H&E ×40) H&E: hematoxylin and eosin

Immunohistochemical staining was performed manually using Coveplates/Slide Rack (Epredia/Thermo Scientific, PHC Holdings Corporation, Portsmouth, NH, USA). The primary antibodies included CD20 (clone L26, Cell Marque; 1:99 µL), BCL-2 (clone EP-17, Cell Marque; 1:99 µL), Ki-67 (clone SP6, Cell Marque; 2:98 µL), and MUM-1 (clone EP190, Bio SB; 2:98 µL). Detection was performed using the Mouse/Rabbit ImmunoDetector DAB HRP Brown kit (Bio SB, Inc., Santa Barbara, CA, USA), a polymer-based horseradish peroxidase detection system using 3,3′-diaminobenzidine as the chromogen. Immunohistochemical analysis demonstrated positivity for CD20, BCL-2, and MUM-1, with a high Ki-67 proliferation index of approximately 80% (Figure 3A-3D), supporting the diagnosis of PCDLBCL-LT.

**Figure 3:**
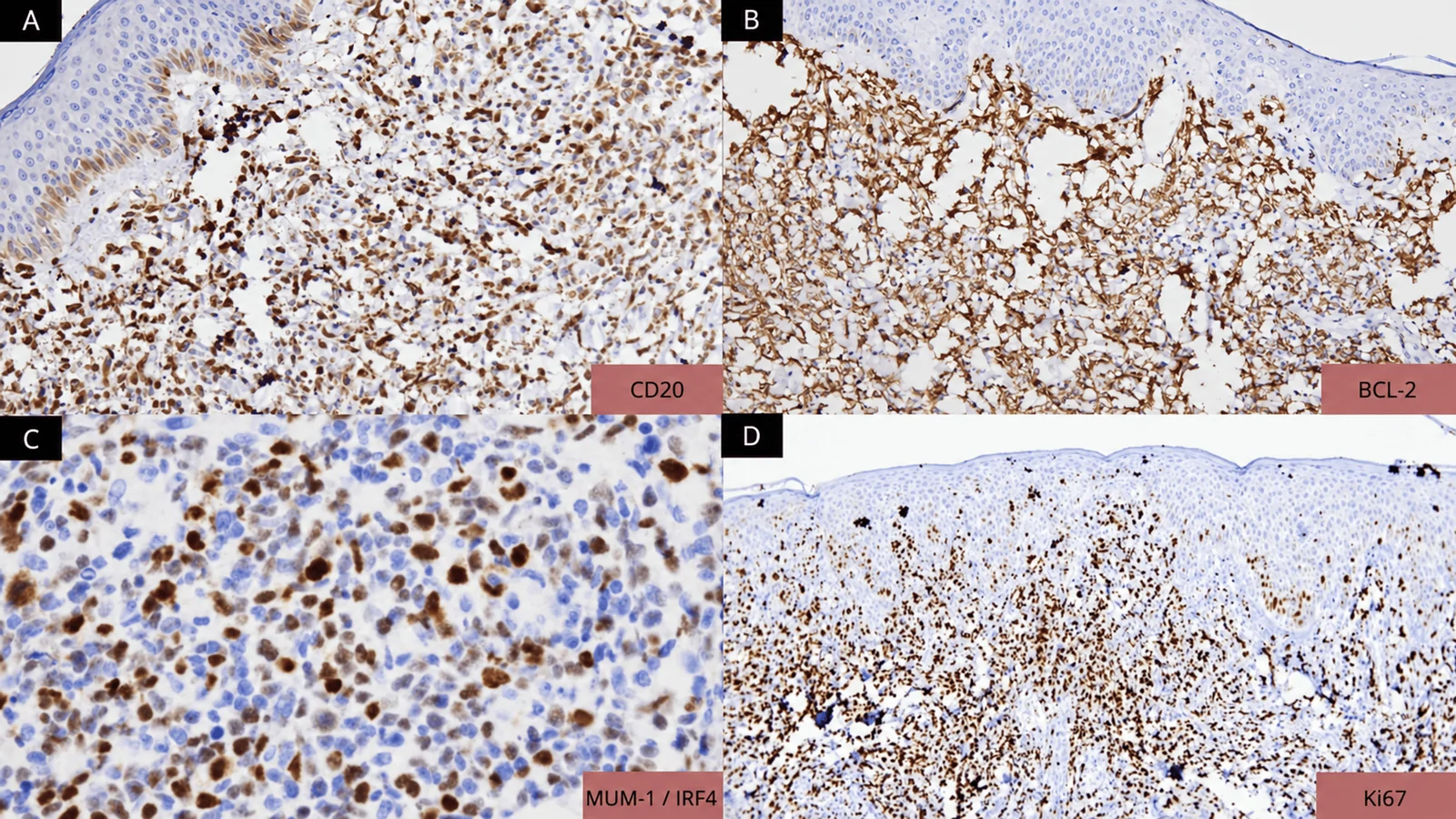
Immunohistochemical findings supporting the diagnosis of PCDLBCL-LT. (A) CD20 immunostaining (×200) showing diffuse membranous positivity in the neoplastic B cells. (B) BCL-2 immunostaining (×200) demonstrating diffuse expression by the tumor cells. (C) MUM-1/IRF4 immunostaining (×400) showing strong nuclear positivity. (D) Ki-67 immunostaining (×100) demonstrating a high proliferative index PCDLBCL-LT: primary cutaneous diffuse large B-cell lymphoma, leg type

Staging workup included laboratory studies showing hemoglobin of 15 g/dL, a platelet count of 322 × 10³/µL, and a serum lactate dehydrogenase level of 152.8 U/L. Serologic testing for hepatitis B and hepatitis C was negative, and HIV testing was nonreactive. Contrast-enhanced and non-contrast computed tomography of the neck, chest, abdomen, and pelvis, as well as positron emission tomography-computed tomography, showed no evidence of extracutaneous disease at diagnosis. A bone marrow biopsy obtained from the right iliac crest also showed no evidence of lymphomatous involvement. Based on the clinical, histopathological, immunohistochemical, and staging findings, a diagnosis of PCDLBCL-LT was established.

The patient received six cycles of R-CHOP immunochemotherapy at 21-day intervals, with dosing calculated based on a body surface area of 1.82 m², followed by consolidative radiotherapy directed to the cutaneous lesion. Despite this treatment, bone marrow involvement was documented only two months after completion of R-CHOP (Figure 4A-4C), demonstrating early extracutaneous progression and subsequent systemic disease. The patient died seven months after bone marrow involvement was documented.

**Figure 4:**
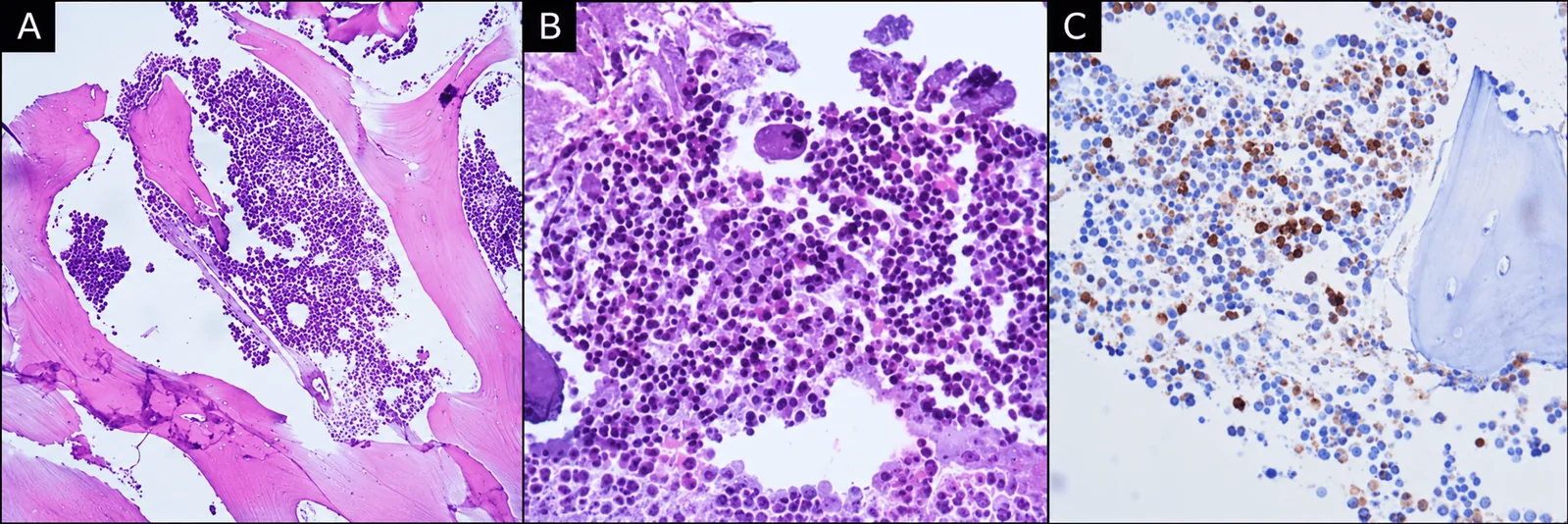
Bone marrow biopsy demonstrating marrow involvement by PCDLBCL-LT. (A) H&E ×40 showing a low-power view of the bone marrow biopsy. (B) H&E ×400 demonstrating diffuse infiltration by atypical lymphoid cells. (C) CD20 immunohistochemistry ×400 showing diffuse membranous positivity in the neoplastic B-cell infiltrate PCDLBCL-LT: primary cutaneous diffuse large B-cell lymphoma, leg type, H&E: hematoxylin and eosin

## Discussion

According to the 2018 World Health Organization-European Organization for Research and Treatment of Cancer classification, primary cutaneous B-cell lymphomas are classified into three main types: primary cutaneous follicle center lymphoma, primary cutaneous marginal zone lymphoma, and PCDLBCL-LT [1,2,5]. The latter represents the most aggressive form of cutaneous B-cell lymphomas and is defined by a dense infiltrate of immunoblasts and centroblasts with round nuclei and expression of CD20, MUM1, and BCL2 [1,2,4,5].

Clinically, it presents as erythematous or violaceous nodules or plaques, predominantly affecting the lower extremities in more than 70% of cases; however, it may also involve other sites such as the scalp, upper extremities, and trunk [2,3]. Bilateral lower-extremity involvement and multiple cutaneous lesions have been associated with poor prognosis [4,5].

Although PCDLBCL-LT predominantly affects elderly women, current evidence does not support male sex as an adverse prognostic factor. In a multicenter study of 60 patients, sex did not significantly affect disease-specific survival, whereas leg localization and multiple skin lesions were independent predictors of poorer prognosis [4]. Therefore, although the occurrence of PCDLBCL-LT in our male patient represents a less common demographic presentation, his aggressive clinical course cannot be attributed to sex alone.

Regarding treatment, survival has improved significantly with immunochemotherapy, particularly the R-CHOP regimen (rituximab combined with anthracycline-based chemotherapy), which is considered first-line therapy, especially in patients with multiple cutaneous tumors [3,4]. Our patient received six cycles of R-CHOP followed by consolidative radiotherapy.

Grange et al. reported an increase in five-year disease-specific survival from 46% to 66% after introducing rituximab [3]. However, Nikolaou et al. noted that despite high initial complete response rates with immunochemotherapy, relapse remains a major challenge in PCDLBCL-LT [5].

Spontaneous regression of PCDLBCL-LT is exceedingly rare, with only six cases reported in the literature, typically occurring within three months after biopsy and in patients with a clinical history of less than one year [6]. These reports consistently describe a marked T-cell infiltrate within the tumor microenvironment, which may modulate the disease course [6,7]. In contrast, our patient showed no spontaneous regression and developed early extracutaneous progression with bone marrow involvement, highlighting the markedly different and more aggressive clinical course in this case.

## Conclusions

This case highlights the aggressive clinical behavior of PCDLBCL-LT, including its potential for early extracutaneous dissemination despite standard treatment. Although PCDLBCL-LT predominantly affects elderly women, our patient was an elderly man who exhibited persistent cutaneous disease despite six cycles of R-CHOP and consolidative radiotherapy, followed by bone marrow involvement only two months after completion of R-CHOP. This clinical course emphasizes the importance of comprehensive staging and close follow-up, even after standard immunochemotherapy. Furthermore, the absence of molecular characterization in this case highlights the potential value of prognostic biomarkers and molecular studies for identifying aggressive disease and supporting more individualized therapeutic strategies.
